# A single-cell transcriptomic atlas of immune cells in Wilson disease identifies copper-specific immune regulation

**DOI:** 10.1016/j.isci.2025.112450

**Published:** 2025-04-17

**Authors:** Shuya Wang, Xianlei Sun, Qingxuan Xin, Jianxiang Shi, Jin Li, Huilin Zhang, Mengjiao Xue, Fanxiang Yin, Zan Qiu, Xiaoqian Wang, Nannan Sun, Yingmei Li, Yaoyao Chen, Liyan Fu, Chaoqi Li, Shaohua Yan, Xian Zhao, Bolin Jue, Yanxia Gao, Baohong Yue, Bo Qin, Yong Jiang, Rongqun Guo

**Affiliations:** 1 Department of Blood Transfusion, The First Affiliated Hospital of Zhengzhou University, Zhengzhou, Henan, China; 2 Translational Medical Center, The First Affiliated Hospital of Zhengzhou University, Zhengzhou, Henan, China; 3 Basic Medical Research Center, Academy of Medical Sciences, Zhengzhou University, Zhengzhou, Henan, China; 4 Department of Laboratory Medicine, The First Affiliated Hospital of Henan University of Chinese Medicine, Zhengzhou, Henan, China; 5 Precision Medicine Center, Henan Institute of Medical and Pharmaceutical Sciences, Zhengzhou University, Zhengzhou, Henan, China; 6 Department of Hematology, The First Affiliated Hospital of Zhengzhou University, Zhengzhou, Henan, China; 7 Department of Laboratory Medicine, The First Affiliated Hospital of Zhengzhou University, Zhengzhou, Henan, China; 8 Xinxiang Medical University College of Basic Medical Sciences, Xinxiang, Henan, China; 9 Department of Emergency Medicine, The First Affiliated Hospital of Zhengzhou University, Zhengzhou, Henan, China; 10 State Key Laboratory of Antiviral Drugs, The First Affiliated Hospital, Zhengzhou University, Zhengzhou, Henan, China; 11 Henan Key Laboratory of Critical Care Medicine, Department of Emergency Medicine, The First Affiliated Hospital, Zhengzhou University, Zhengzhou, Henan, China; 12 Institute of Infection and Immunity, Henan Academy of Innovations in Medical Science, Zhengzhou, Henan, China

**Keywords:** Biological sciences, Immunology, Immune system evolution, Immune system, Immune response, Transcriptomics

## Abstract

Wilson disease (WD) is caused by mutations of the copper-transporting gene, *ATP7B*, leading to abnormal copper metabolism. A better characterization of WD is essential in understanding the effects of excess copper and how it disrupts immune regulation and hematopoietic development. Furthermore, the exploration of the relationship between copper-mediated proliferation or cuproptosis and immune regulation is critical for developing new immune therapies. Therefore, we performed single-cell RNA sequencing (scRNA-seq) on peripheral blood mononuclear cells (PBMCs) to develop an atlas of the immune landscape. Cells were clustered into several immune subsets, and cuproptosis-associated genes were assessed. Differential expression analysis was performed to identify WD-specific signatures by comparing transcriptome profiles of patients with WD with HDs. Excess copper impaired immune homeostasis and hematopoietic development. Then, we developed a map of the immune landscape of patients with WD. Excess copper is involved in the metabolic reprogramming of immune cells, such as glycolysis in CD14^+^ monocytes. We found that the antigen processing-related pathway is dysregulated in immune cells of patients with WD. Our study revealed that abnormal copper concentration influences the expression of HLA-I and HLA-II molecules. It is noteworthy that a high concentration of intracellular copper differs significantly from the high concentration of extracellular copper. We have also identified a gene set of neurologic abnormalities, which were dysregulated in PBMCs of patients with WD. We also observed abnormal expression of cuproptosis-associated genes in proliferating or malignant cells, providing new insights into the application of cuproptosis in cancer treatment.

## Highlights

• We constructed an atlas of immune cells in Wilson’s disease utilizing scRNA-seq analysis
• Copper-specific immune regulation was identified with the involvement of antigen processing
• Excess copper alters the biological process of malignant cells

## Graphical abstract

**Figure:**
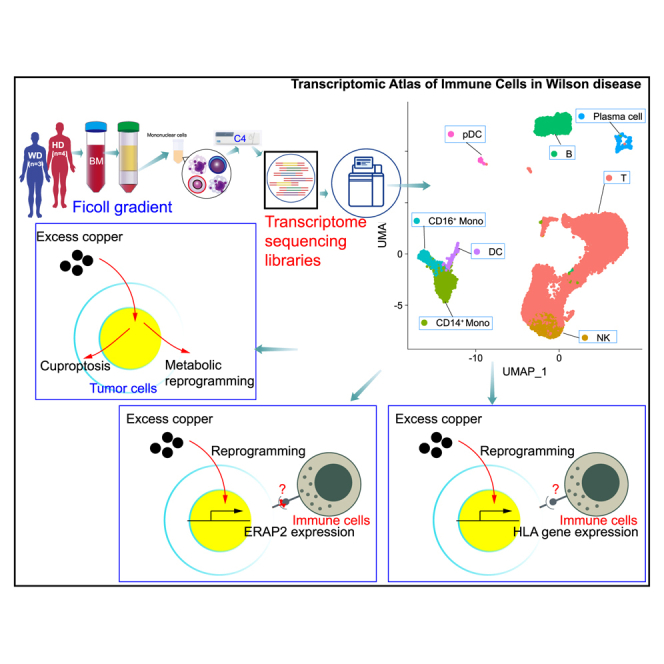

## Introduction

Wilson disease (WD) is a hereditary condition characterized by impaired copper metabolism, resulting from mutations in the P-type ATPase ATP7B gene.^1^ Abnormal copper metabolism primarily manifests as liver disease and neuropsychiatric disorders, which are the prevailing symptoms. Hypersplenism, which may arise as a consequence of cirrhosis caused by WD, could cause leukopenia and thrombocytopenia.^2^ Adequate copper levels play a crucial role in maintaining the proper functioning of mitochondria and copper-dependent enzymes, namely PDE3B, MEK1, MEK2, ULK1, and ULK2. Consequently, any disturbances in copper metabolism can negatively impact the homeostasis of lipolysis, cell growth/proliferation, and autophagy.^3^ Hepatocyte death is a consequence of copper-mediated disruption of iron-sulfur (FeS) containing enzymes.^4^ ATP7B has the capability to translocate from the Golgi apparatus to lysosomes, where it engages in interactions with p62/dynactin. This interaction is responsible for facilitating the polarized exocytosis of lysosomes, thereby enabling the release of surplus copper.^5^ The accumulation of intracellular copper has been found to trigger a unique form of cell death that is dependent on copper, known as cuproptosis.^6^ The exploration of targeting cuproptosis for therapeutic purposes has been recognized as a promising avenue for clinical applications.^7^

Excessive copper levels in individuals diagnosed with WD disrupts metabolic homeostasis.^8^ Undoubtedly, the presence of atypical levels of copper ions serves as a significant player for metabolic reprogramming.^9^ Elevated copper levels have been observed in numerous tumors, suggesting the involvement of copper metabolism in the process of tumorigenesis.^10^ Many genes involved in copper homeostasis have been found, such as *CP*, *CTR1* (*SLC31A1*), *CTR2* (*SLC31A2*), *ATOX1*, *CCS*, *COX11*, *COX17*, *SCO1*, *SCO2*, *COA6*, *SLC25A3*, *ATP7A*, *ATP7B*, *MT1*, *MT2*, *COX1*, *MT*-*CO2*, *SOD1*, *TYR*, *LOXL2*, *DBH*, *AOC3* (*VAP1*), *MAP2K1*, *MAPK2*, *ULK1*/*ULK2*, *PDK1*, *PDE3B*, *UBE2D1*/*2*/*3*/*4*, *H3C1*, *HC14*, *VEGFA*, and *PD1L1* (*CD274*). For instance, the binding of copper to PDK1 and its subsequent activation of AKT in tumorigenesis exemplifies the potential involvement of abnormal copper metabolism in various undiscovered biological pathways, in addition to its role in cuproptosis.^11^

Approximately 5%–6% of individuals diagnosed with WD exhibit hemolytic anemia.^12^ Furthermore, the pathogenesis and progression of this disease may be influenced by dysfunctional immune cells.^13^ Aberrant N2-neutrophil polarization has been documented in animal models of WD.^8^ Immune and inflammatory disorders, such as Crohn’s disease (CD), have been observed in individuals with WD, suggesting that excess copper may also be involved in immune homeostasis.^14^ scRNA-seq is a powerful tool for illustrating cell states at single-cell level.^15^^,^^16^^,^^17^^,^^18^^,^^19^^,^^20^^,^^21^^,^^22^^,^^23^ In this study, we generated a comprehensive cellular atlas of immune cells derived from PBMCs obtained from patients with WD using scRNA-seq analysis. Our findings reveal that copper plays a crucial role as an immune regulator by modulating the expression of the human leukocyte antigen (HLA) complex. Additionally, we have illustrated the gene sets that possess the potential to serve as predictive markers for distinguishing cuproptosis-sensitive or resistant cells.

## Results

### Copper disrupted the equilibrium of hematopoiesis and immune regulation, albeit not through direct cuproptosis

**Figure 1:**
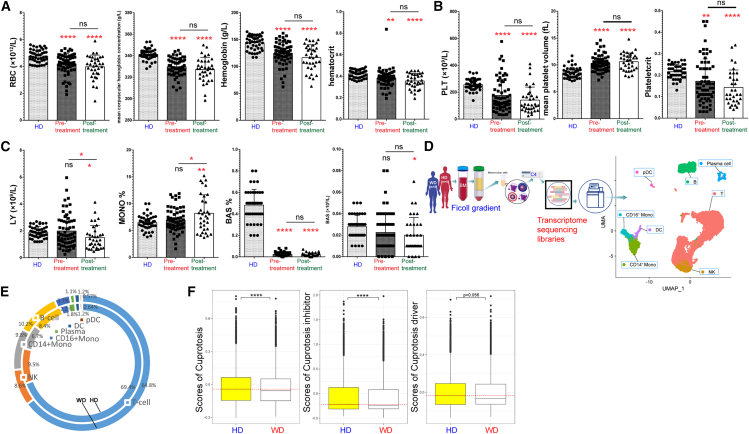
Excessive levels of copper have been found to negatively impact hematopoiesis and immune homeostasis Histograms showing the red blood cell (RBC) count, mean corpuscular hemoglobin concentration, hemoglobin, hematocrit (A), platelet count, mean platelet volume, plateletcrit (B), lymphocyte count, basophil count, and proportion of monocyte and basophil (C) for peripheral blood (PB) from healthy donors (HD) and patients with Wilson disease before or after treatment. (D) UMAP plot shows the distribution of different subsets from HDs and patients with WD. (E) Proportion of different subsets in HD and patients with Wilson’s disease. (F) The scores of cuproptosis-related genes (including both cuproptosis inhibitor and driver genes), cuproptosis inhibitor gene set, and cuproptosis driver gene set.

Patients diagnosed with WD exhibited anemia (Figures 1A and S1A), aligning with previous findings of hemolytic anemia attributed to elevated copper levels.^24^ Moreover, patients with WD may also experience impaired platelet function (Figure 1B), suggesting that WD may be linked to thrombocytopenia, such as immune thrombocytopenia.^25^ Interestingly, immune cells can also be affected, such as lymphocytes, basophils, and monocytes (Figures 1C and S1B). Copper has the ability to selectively target lipoylated proteins involved in the tricarboxylic acid (TCA) cycle, ultimately resulting in the induction of cuproptosis.^6^ In this study, we postulated that cuproptosis is associated with aberrant immune regulation in individuals diagnosed with WD. To validate this hypothesis, scRNA-seq analysis was conducted on PBMCs obtained from patients diagnosed with WD and HDs (Figures S1C and S1D). There were no notable disparities detected in the distribution of various immune subsets among patients with WD and HDs (Figures 1E and S1D). Interestingly, a notable decrease in mRNA expression levels of both the cuproptosis inhibitor and driver gene sets was observed in PBMCs derived from patients, compared to PBMCs obtained from HDs (Figure 1F). Most likely, it can be inferred that copper does not directly elicit abnormal immune activity through cuproptosis.

### The metabolic reprogramming in various immune cell types is induced by excess copper

**Figure 2:**
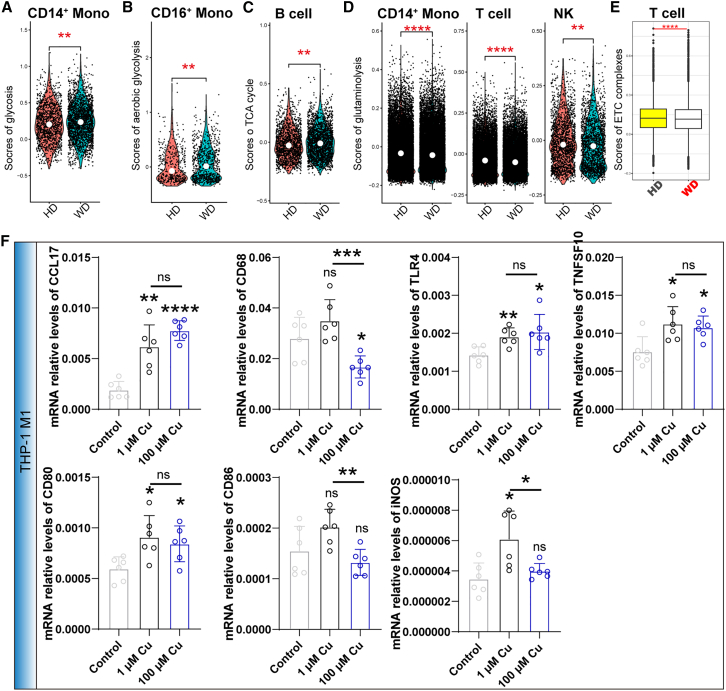
Copper induces metabolic reprogramming of immune cells in patients with WD This reprogramming is evident through the assessment of various metabolic parameters, including glycolysis in CD14^+^ monocytes (A), aerobic glycolysis in CD16^+^ monocytes (B), TCA cycle in B cells (C), glutaminolysis in CD14^+^ monocytes, T cells, and NK cells (D), and ETC complexes in T cells (E). (F) RT-qPCR was performed to quantify the relative mRNA levels of *CCL17*, *CD68*, *TLR4*, *TNFSF10*, *CD80*, *CD86*, and *iNOS* in THP-1-derived M1 macrophages treated with different concentrations of copper.

Copper plays a crucial role in immune cells by serving as both an electron acceptor and donor.^26^ Therefore, it is hypothesized that excess copper exerts an influence on the metabolic profile of PBMCs. Next, the Seurat package’s *AddModuleScore* function was employed to evaluate the expression pattern of various metabolic pathways, including glycolysis, pentose phosphate pathway (PPP), aerobic glycolysis, TCA cycle, glutaminolysis, fatty acid metabolism, and electron transport chain (ETC) complexes.^27^ The study revealed alterations in the metabolic states of PBMCs in individuals diagnosed with WD, specifically affecting the pathways of PPP, glycolysis, fatty acid metabolism, and glutaminolysis (Figure S2A). In addition, various immune subsets exhibit distinct metabolic preferences, such as the notable upregulation of glycolysis-associated genes in monocytes and DC (Figure S2B). The presence of an excessive amount of copper triggers a significant metabolic reprogramming in various immune subtypes (Figure S2C). The glycolysis-associated genes were upregulated in CD14^+^ monocytes from patients with WD (Figure 2A). The activation of classical monocytes is reliant upon glycolytic metabolism, which encompasses the activation of p38 MAPK and the accumulation of reactive oxygen species (ROS).^28^ Furthermore, there was a notable elevation in the levels of aerobic glycolysis in CD16^+^ monocytes derived from individuals diagnosed with WD (Figure 2B). Aerobic glycolysis is important for the immune response of M1 macrophages, T cells, and intermediate monocytes.^29^^,^^30^^,^^31^ The upregulation of TCA cycle-associated genes was observed in B cells derived from patients with WD, suggesting that copper-mediated alterations in the TCA cycle may impact humoral immunity.^32^ Additionally, our study demonstrated that an overabundance of copper exerts inhibitory effects on the expression of genes associated with glutaminolysis in CD14^+^ monocytes, T cells, and NK cells (Figure 2D). The expression pattern of ETC complexes, particularly complex I, exhibited alterations in PBMC derived from patients with WD (Figure S3A). Plasma cells exhibit a significantly higher expression of mRNA associated with ETC complexes compared to other lineages (Figure S3B). Notably, excess copper primarily affects the ETC complexes of T cells, particularly complex I/IV (Figures 2E, S3C, and S3D). Consequently, copper has the potential to induce metabolic reprogramming in immune subsets among patients with WD.

To further investigate the functions of copper in immune regulation, we treated THP-1-derived M1 macrophages with copper (100 nM/mL) and a competitive inhibitor of glucose metabolism, 2-DG. Interestingly, low doses of extracellular copper did not have a great effect on the immune function of macrophages compared to 2-DG (Figure S4A). We then increased the concentrations of copper and found that high doses of copper increased the expression levels of proinflammatory genes, including CCL17, CD68, TLR4, TNFSF10, CD80, CD86, and iNOS (Figures 2F and S4B). These results indicated that the accumulated high concentration of copper may promote pathological changes of the liver mediated by the hyperactivation of inflammation.

### Excessive presence of copper has a detrimental impact on the biological process of antigen processing in patients with Wilson disease

**Figure 3:**
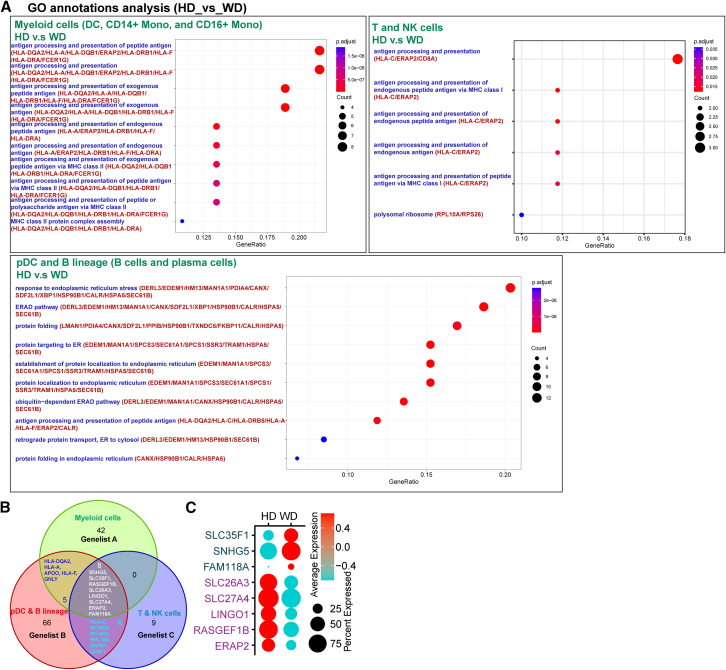
There is a correlation between an excessive presence of copper and the process of antigen processing (A) Dot plot visualization of GO analysis based on DGEs in myeloid cells, pDC/B lineages, and T/NK cells between HD and patients with Wilson’s disease. (B) Venn plot visualization of common DGEs in different immune subsets. (C) Dot plot visualization of common DGEs in PBMCs from different groups (HD and patients with WD).

To investigate the impact of copper, we performed a comparative analysis of differentially expressed genes (DEG) in myeloid cells, pDC/B lineages, and T/NK cells from both HDs and patients with WD (Figure 3A). Remarkably, the enrichment of antigen processing-associated genes provides novel insights into the investigation of the correlation between WD and immune dysregulation (Figure S5A).^33^ The presence of HLA-DR has been found to be correlated with markers of disease severity, inflammatory mediators, and patient outcomes in individuals experiencing acute liver failure,^34^ which suggests that impaired antigen processing may play a role in the development of liver failure in patients with WD.

In this study, a total of eight distinct genes (*SNHG5*, *SLC35F1*, *RASGEF1B*, *SLC26A3*, *LINGO1*, *SLC27A4*, *ERAP2*, *FAM118A*) were successfully identified within three prominent immune subsets (Figures 3B and S5B). It was found that SNHG5 was involved in neurological disorders,^35^^,^^36^ SLC35F1 was involved in neurological disorders, developmental and epileptic encephalopathies resembling Rett syndrome.^37^ RASGEF1B was linked to neurodevelopmental and neuromuscular abnormalities in 4q21.22 syndrome.^38^^,^^39^ SLC26A3 was associated with both congenital chloride diarrhea and Alzheimer’s disease (AD).^40^^,^^41^ Furthermore, SLC27A4 was found to be involved in neurological disorders as well as Ichthyosis prematurity syndrome.^42^ LINGO1 has been implicated in abnormal axonogenesis in patients with AD.^43^ ERAP1, classified as one of the antigen processing aminopeptidases, has involved in classical MHC-I-opathies.^44^ The gene, FAM118A, has been found to be associated with both ankylosing spondylitis and glioblastoma, as indicated by previous studies.^34^^,^^45^^,^^46^^,^^47^ Collectively, these data not only support the results of copper-mediated regulation of antigen processing (Figure S5C) but also present a useful gene set for predicting the progression of neurological abnormalities (Figure 3C).

### Copper exerts a significant influence on the immune response by modulating the MHC

**Figure 4:**
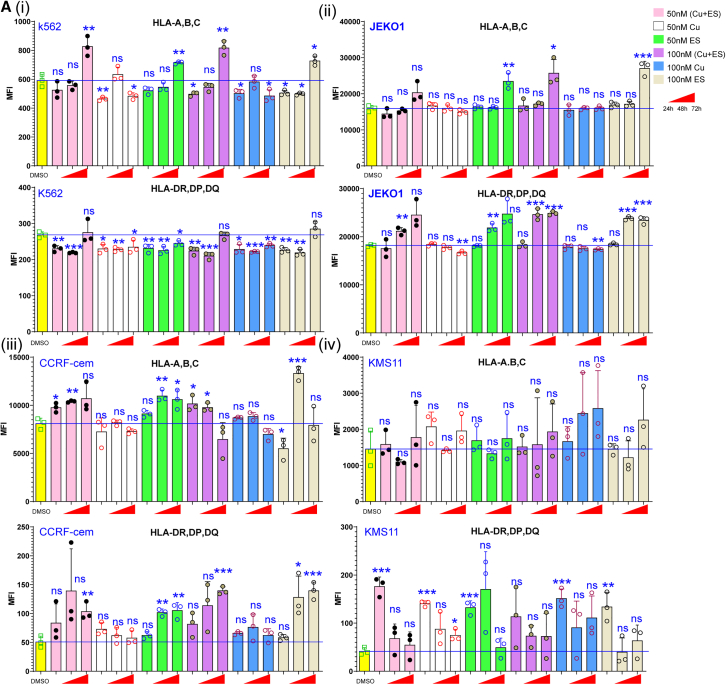
The function of intracellular copper differs significantly from that of extracellular copper (A) The bar diagram shows the MFI for HLA-A, B, C and HLA-DR, DP, DQ expression on K562 cells (i), JEKO-1 cells (ii), CCRF-cem cells (iii), and KMS11 cells (iv) treatment with the indicated concentrations of copper and/or ES for 24h, 48h, and 72h. Bar graphs show the mean ± SD.

MHC molecules play a prominent role in maintaining immune tolerance and homeostasis under normal physiological circumstances. To enhance the comprehensibility of our findings, we subjected various cell lines to copper treatment and/or the application of Cu ionophores, specifically elesclomol (ES). Time-dependent MHC expression patterns can be observed in 293T cells after treatment with copper and/or ES (Figure S6A). The downregulation of HLA-A, B, and C and HLA-DR, DP, and DQ was observed in monocyte-like cell line THP-1 totally (Figure S6B), and similar results can be gotten from chronic myelogenous leukemia (CML) cell line K562 in less than 48 h after treatment with ES with or without copper (Figure 4Ai). Notably, the expression of surface HLA molecules in the mantle cell lymphoma (MCL) cell line JeKo-1 was found to be influenced by ES, regardless of the presence or absence of exogenous copper (Figure 4A(ii)). These findings were also observed in human T lymphoblasts CCRF-CEM (Figure 4A(iii)) and the immortalized T lymphocyte cell line Jurkat (Figure S6C). Interestingly, we found that excess copper increased HLA-DR, DP, and DQ surface proteins in less than 24 h, but decreased with an extension of culture time (Figure 4A(iv)). Remarkably, the administration of a solitary copper supplement exhibited a reduction in the protein levels of surface human leukocyte antigen (HLA) molecules in THP-1, JEKO-1, CCRF-CEM, Jurket, and NK92-MI cell lines (Figure S6D). Evidently, these findings suggest that intracellular and extracellular copper exert disparate influences on the modulation of surface MHC molecules. Additionally, different cell lines presented diverse effects on the expression of MHC molecules after being treated with copper and/or its ionophores.

### Cuproptosis has the potential to selectively target hematopoietic cells that are proliferating or exhibit progenitor-like characteristics

**Figure 5:**
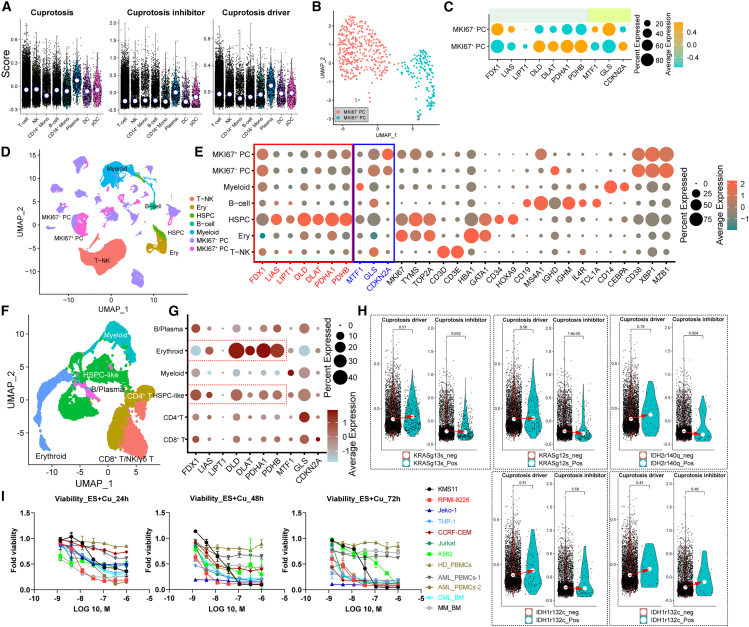
The genes associated with cuproptosis are abnormally expressed in cells undergoing proliferation or exhibiting malignancy (A) The scores of cuproptosis-associated genes (cuproptosis inhibitor + driver), cuproptosis inhibitor gene set, and cuproptosis driver gene set in different immune subsets from HDs and patients with WD. (B) UMAP projection of plasma cells from PBMCs of both HDs and patients with WD. (C) Dot plots show the expression of cuproptosis-associated genes. (D) UMAP projection of bone marrow cells from patients with MM. (E) Dot plots show the expression of cuproptosis-associated and lineage-specific genes. (F) UMAP projection of bone marrow cells from patients with AML or MDS. (G) Dot plots show the expression of cuproptosis-associated genes. (H) The scores of cuproptosis inhibitor-associated genes and cuproptosis driver-associated genes in mutation-carrying HSPC-like cells and their counterparts from patients with AML or MDS. (I) Quantitation of cell death of primary cells and cell lines after 24, 48, and 72 h of treatment with ES and copper.

Plasma cells were observed to exhibit elevated expression levels of cuproptosis-associated genes in comparison to other lineages (Figure 5A). To discern the susceptible subtypes of plasma cells, a reclustering approach was used, resulting in the identification of two major subtypes, namely MKI67^+^ and MKI67^-^ plasma cells (Figure 5B). The expression levels of cuproptosis driver-associated genes are higher in proliferating MKI67^+^ plasma cells, while the expression levels of cuproptosis inhibitor-associated genes are lower in comparison to MKI67^-^ plasma cells (Figure 5C). This distinctive phenotype observed in MKI67^+^ plasma cells prompted us to investigate the potential relationship between cuproptosis and multiple myeloma (MM) (Figure S7A). In addition to our investigation of scRNA-seq analysis in patients with MM (Figure 5D), we have discovered that plasma cells exhibit significant expression of cuproptosis-associated genes (Figure 5E). Remarkably, we have also observed elevated mRNA expression levels of cuproptosis driver-associated genes in hematopoietic stem/progenitor cells (HSPC) (Figure S7B), which prompted us to evaluate the impact of cuproptosis on hematologic malignancies. Subsequently, we conducted targeted scRNA-seq on bone marrow cells obtained from individuals diagnosed with acute myeloid leukemia (AML) or myelodysplastic syndromes (MDS),^27^ as illustrated in Figure 5F. Consistent with results from patients with MM, HSPC-like cells from patients with hematologic malignancies expressed cuproptosis-associated genes at high levels compared with lymphoid cells and mature myeloid cells (Figure 5G). Surprisingly, we found erythroid lineages show the cuproptosis sensitivity based on the high expression of cuproptosis driver-associated genes and low expression of cuproptosis inhibitor-associated genes, which indicates that excess copper may impair the erythroid development as a result of anemia in patients with WD (Figure 5G). Surprisingly, we found erythroid lineages show the cuproptosis sensitivity based on the high expression of cuproptosis driver-associated genes and low expression of cuproptosis inhibitor-associated genes, which indicates that excess copper may impair the erythroid development as a result of anemia in patients with WD. Interestingly, our investigation revealed that erythroid lineages displayed susceptibility to cuproptosis, as evidenced by their heightened expression of cuproptosis driver-associated genes and diminished expression of cuproptosis inhibitor-associated genes. This suggests that an excessive accumulation of copper may impede erythroid development, consequently leading to anemia in patients with WD. Significantly, our study revealed that the HSPC-like malignant cells harboring mutations (*KRAS*^*G13S*^, *KRAS*^*G12S*^, *IDH2*^*R140Q*^*,* and *IDH1*^*R132C*^) exhibited elevated expression levels of cuproptosis driver-associated genes and diminished expression levels of cuproptosis inhibitor-associated genes compared to their corresponding counterparts (Figure 5H). Similar findings were observed in both erythroid lineages and mature myeloid lineages (Figure S7C). Together, these compelling findings suggest the potential application of cuproptosis as a therapeutic approach for hematologic malignancies.

To test the potential of targeting hematologic malignancies, we then exposed several cell lines (KMS11, RPMI8226, THP-1, CCRF-cem, and Jurkat) and primary human cells (PBMCs from HD and a patient with AML) for a different duration compared with the copper and/or ES (Figures 5I and S8). On its own, copper did not decrease the cell viability, but both ES on its own and the combination of ES and copper acutely decreased the cell viability (Figure S7D). Interestingly, KMS11 cells showed strong resistance to cuproptosis, consisting of the expression pattern of cuproptosis-associated genes in primary plasma cells. Together, our results would provide the guidance of cuproptosis-mediated anti-tumor, avoiding ineffective treatment. We associated cuproptosis with proliferating or malignant cells.

### Copper can be used to target leukemia cells with the IDH2^R140Q^ mutation

**Figure 6:**
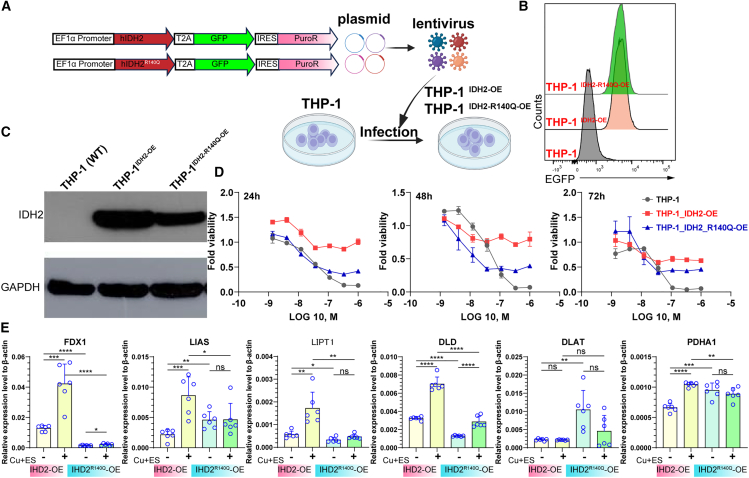
Combination of excess copper and IDH2^R140Q^ mutation suppresses viability of leukemia cells (A) Schematic diagram of *IDH2* and *IDH2*^R140Q^ overexpression lentiviral vectors. (B) Flow cytometry measurements of GFP in wild type THP-1, THP-1-IDH2^OE^, and THP-1-IDH2^R140Q−OE^ cell lines. (C) Western Blot analysis of overexpression of IDH2 in wild type THP-1, THP-1-IDH2^OE^, and THP-1-IDH2^R140Q−OE^ cell lines. (D) Quantitation of cell death of wild type THP-1, THP-1-IDH2^OE^, and THP-1-IDH2^R140Q−OE^ cell lines after 24, 48, and 72 h of treatment with copper. (E) RT-qPCR was performed to quantify the relative mRNA levels of *FDX1*, *LIAS*, *LIPT1*, *DLD*, *DLAT*, and *PDHA1* in THP-1-IDH2^OE^, and THP-1-IDH2^R140Q−OE^ cell lines treated with different concentrations of copper combination with ES (0.1 μM) for 12 h.

IDH2 mutation and copper are both involved in metabolic reprogramming, so we tried to establish leukemia cells carrying IDH2^R140Q^ mutation for examining the potential effects under excess copper conditions (Figure 6A). We obtained leukemia cell lines overexpressing wide-type IDH2 and IDH2^R140Q^ (Figures 6B and 6C). After 48 h of excess copper (1 × 10^−9^∼1 × 10^−7^ M) treatment, a combination of copper and IDH2^R140Q^ mutation robustly inhibited the viability of leukemia cells compared to wild type THP-1 and THP-1 with wild type IDH2 overexpression (Figures 6D and S9). Surprisingly, IDH2^R140Q^ mutation changed the expression patterns of cupropotosis-associated genes, including *FDX1*, *LIAS*, *LIPT1*, *DLD*, *DLAT*, and *PDHA1* (Figure 6E), which indicated that the sensitivity of tumor cells to cuproptosis depends on the types of genetic mutations. Taken together, copper-associated therapeutics agents can be used to develop new strategies for targeting hematological malignancies, such as leukemia with IDH2 mutation.

### Copper alters the sensitivity of tumor cells to immune cell-mediated killing

**Figure 7:**
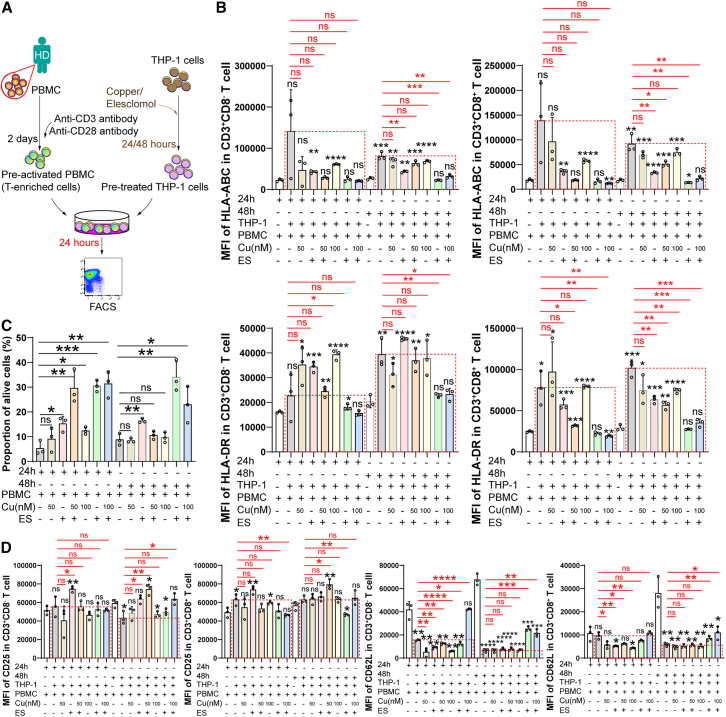
Effects of excess copper on immune responses of tumor cells and immune cells (A) Schematic showing the workflow used to establish a co-culture system of treated THP-1 cells and pre-activated T cells. (B) The surface expression levels of HLA-ABC and HLA-DR in T cells, as detected by flow cytometry. (C) Flow cytometry measurements of the alive proportions of THP-1 cells. (D) The surface expression levels of CD25 and CD62L in T cells, as detected by flow cytometry.

To investigate whether copper treatment influenced immune regulation, we cocultured copper/ES-treated THP-1 cells with T cells (Figure 7A). Flow cytometry analysis of HLA-related surface proteins revealed ES-treated THP-1 inhibited the upregulation of HLA-A/B/C and HLA-DR (Figure 7B). HLA-DR and HLA class I (ABC) can be upregulated in T cells during an immune response.^48^^,^^49^ Surprisingly, ES-treated THP-1 cells showed high survival rates after co-incubation with T cells (Figure 7C). Furthermore, ES-treated THP-1 cells (48 h) did not drive the downregulation of CD62L in T cells (Figure 7D), suggesting that T cells were not being activated effectively. Together, excessive copper can not only directly affect the cell fate of tumor cells, but also indirectly change the responses of immune cells to tumor cells.

## Discussion

Excess copper induces the degradation of XIAP protein, increasing apoptosis.^50^ Interestingly, excess copper can bind directly to amyloid-β (Aβ) peptides with high affinity, which leads to increased neurotoxicity in patients with Alzheimer’s disease because of the aggregation of Aβ, hydroxyl radical (·OH) production, and oxidative damage.^7^ Excess copper may induce cell death, contributed by apoptosis, caspase-independent cell death, and accumulation of ROS.^51^^,^^52^^,^^53^^,^^54^ Surprisingly, copper can enhance ferroptosis by promoting the autophagic degradation of GPX4.^55^ Toxic mitochondrial protein aggregation is a result of excess intracellular copper,^56^ hence the need to identify the effects of the combination of copper ionophore and proteasome inhibitors in the future. Our study showed *IDH1*/*2* and *KRAS* mutation-carrying malignant cells presented dysregulated cuproptosis-associated genes, which may help to target tumor cells more precisely and avoid damage to beneficial cells (such as cytotoxic cells and normal HSPCs).^57^ Unquestionably, cuproptosis may be a critical target of cancer treatment.

Alternatively, the patients with WD may initially present with psychiatric symptoms.^58^ Parkinson's disease, lupus nephritis, cardiac involvement, subcutaneous lipomas, multiple sclerosis, calf muscle involvement, and IgA nephropathy, maybe associated with WD, which have been reported.^59^^,^^60^^,^^61^^,^^62^^,^^63^^,^^64^ These interesting cases indicated that autoimmune or inflammatory diseases may be a result of excess copper. Importantly, our findings showed that abnormal copper metabolism is associated with dysfunctional MHC expression. Our results indicated that the expression pattern of ERAP2 was impaired in patients with WD. Downregulated ERAP2 might weaken the peptidome presented by HLA-E and impair HLA-E-NKG2A checkpoint, which further enhances NK and CD8^+^ T cells cytotoxicity.^65^ Furthermore, endoplasmic reticulum stress (ERS)-associated pathways were changed in B lineage from patients with WD. ERS is a critical immune signaling and loss of endoplasmic reticulum homeostasis is involved in the pathogenesis of HLA-associated disorders.^66^^,^^67^ Interestingly, copper can upregulate PD1L1, leading to cancer immune evasion.^68^ Many pharmacological agents, such as D-Penicillamine, Trientine, Tetrathiomolybdate, ATN-224, Disulfuram, Elesclomol, Diacetylbis (N^4^-methylthiosemicarbazonato) copper (II), Glyoxalbis (N4-methyl-3-thiosemicarbazonato) copper (II), Clioquinol, ^64^Cu, and DC_AC50, have been developed to target copper metabolism.^10^ Therefore, regulating the concentration of copper may be applied into not only cuproptosis-associated cancer treatment but also immune regulation.

Penicillamine, a well-established antirheumatic medication, has been approved for the treatment of Wilson's disease in adults. Several studies have suggested a potential relationship between penicillamine and immune regulation.^69^^,^^70^ Furthermore, while the patients with Wilson disease included in our study have been clinically diagnosed, the effects of various ATP7B mutations, such as P992L, A874P, R778L, and c.1543 + 1G>T, may differ.^71^^,^^72^ Additionally, sex-biased immune compartments, encompassing immune gene expression and immune cell populations, as well as aging-driven immunosenescence, have been identified in numerous studies and may also impact our findings.^73^^,^^74^^,^^75^^,^^76^ Due to ethical considerations in medical research and the scarcity of patients with Wilson disease, our study utilized specimens from patients who had previously received treatment and had an established diagnosis. Consequently, we cannot exclude the possibility of confounding variables related to treatment, sex, or age.

Our results also showed that many psychiatric disease-associated remarkable genes, were upregulated (*SLC35F1*, *SNHG5*, and *FAM118A*) or downregulated (*SLC26A3*, *SLC27A4*, *LINGO1*, and *RASGEF1B*) in PBMCs of patients with WD. These findings lead us to be uncertain as to whether these genes can be developed into specific clinical indexes for monitoring the degree of neuropathy in patients with WD.

Collectively, we establish a cellular atlas of immune cells from patients with WD. Interestingly, cuproptosis does not seem to be present in PBMCs from patients with WD, basing the scRNA-seq analysis. Notably, abnormal HLA expression is observed. Our *in vitro* studies suggest that extracellular and intracellular excess copper present different effects on the expression of MHC protein. Interestingly, we found that proliferating plasma cells and HSPCs express cuproptosis-associated genes at high levels, and tumor cell lines are sensitive to excess extracellular copper but not normal PBMCs. Moreover, our study presents a neural disease-associated gene set, which can be regarded as an index of neuropathy.

The data presented in this study establish the initial framework of the cellular atlas of immune cells from patients with WD, while also highlighting the role of copper in metabolic reprogramming. A comparative analysis between patient-derived PBMCs and those from HDs reveals a distinct set of DEGs associated with neurologic disorders, including *SNHG5*, *SLC35F1*, *RASGEF1B*, *SLC26A3*, *LINGO1*, *SLC27A4*, and *FAM118A*. These findings offer novel insights into the prediction of neurologic changes induced by WD. Significantly, our study revealed that the antigen processing-associated pathways were impaired in immune cells of patients with WD. Additionally, we discovered that varying concentrations of intracellular and extracellular copper had distinct effects on the expression of surface MHC molecules, suggesting the potential for copper-mediated immune regulation. Despite cuproptosis not appearing to be involved in the cell fates of most immune subsets in patients with WD, there were notable expression patterns of cuproptosis-associated genes in proliferating plasma, HSPCs, and malignant cells. This observation highlights the potential of cuproptosis in the treatment of tumors. Our future objective is to devise novel therapeutic approaches for immune regulation in the context of tumor treatment, autoimmune diseases, and organ/cell transplantation.

### Limitations of the study

In this study, we show that excess copper or dysfunctional copper metabolism alters the biological process of antigen processing and presentation, involving HLA-related genes and ERAP2. However, the molecular mechanisms underlying this alteration remain poorly characterized. Our study has several limitations, which limit us to understand the alteration of HLA-associated biological processes clearly. First, due to the limitations of clinical ethical considerations, we are unable to obtain samples from patients without treatment. This unavoidable bias may affect our results, which have been demonstrated by measuring copper concentration in PBMCs from HDs and patients with WD (Figure S10). Does D-penicillin also affect the biological process of antigen processing? In the future, we will use a mouse model to overcome this. Second, we have not conducted *in vivo* studies on whether excess copper will affect the HLA-associated changes in tumor cells and whether such changes are detrimental or beneficial. Furthermore, excess copper not only affects tumor cells but also immune cells, further possibly overburdening the already taxed immune cells.

## Resource availability

### Lead contact

Inquiries and requests for resources should be directed to and will be fulfilled by the lead contact, Dr. Rongqun Guo (guorq2007@163.com).

### Materials availability

All cell lines used in this study will be made available on request to the lead contact; however, the requestor will cover shipping costs. This study did not generate new unique reagents.

### Data and code availability

• Data. The raw data for scRNA-seq have been deposited in the Genome Sequence Archive in the National Genomics Data Center, China National Center for Bioinformation/Beijing Institute of Genomics, Chinese Academy of Sciences (GSA-Human: HRA006240), which are publicly accessible at https://ngdc.cncb.ac.cn/gsa-human. And the processed matrix files can be found in *Figshare* (https://doi.org/10.6084/m9.figshare.27977781). All data reported in this article will be shared by the lead contact upon request.
• Code. This article does not report original code.
• Any additional information required to reanalyze the data reported in this article is available from the lead contact upon request.

## Acknowledgments

This work was supported by the 10.13039/501100001809National Natural Science Foundation of China (82100240, RQ. Guo), 10.13039/501100012166National Key R&D Program of China (2023YFF0714402, B. Qin), the Key scientific research projects of colleges and universities in Henan Province (25B310003, SY. Wang; 25A320001, RQ. Guo; 22A320016, RQ. Guo), Joint Co-construction Project of Henan Medical Science and Technology Research Plan (LHGJ20200280, RQ. Guo; LHGJ20210322, FX. Yin), Provincial and Ministry Joint Co-construction Project of Henan Medical Science and Technology Research Plan (SBGJ202103045, RQ. Guo; SBGJ202302067, B. Qin), Key Research and Development and Promotion Project of Henan province (212102310755, RQ. Guo), Science Fund Program of Henan for Distinguished Young Scholars (232300421049, RQ. Guo), national talent cultivation project on first-class academic discipline construction (clinical medicine) of Zhengzhou University, Science and Technology Research Project of Henan Provincial Science and Technology Department (242102311198, B. Qin), and National Natural Science Foundation of Henan Province (212300410269, SY. Wang).

We also acknowledge the assistance from the Translational Medical Center at The First Affiliated Hospital of ZZU.

## Author contributions

SY. Wang, RQ Guo, B. Qin, NN. Sun, and QX. Xin collected BM/PB samples and clinical data. RQ. Guo, SY. Wang, Y. Jiang, and XL. Sun designed the experiments. XL. Sun, JX. Shi, RQ. Guo, J. Li, FX. Yin, Z. Qiu, LY. Fu, XQ. Wang, YY. Chen, YM. Li, QX. Xin, HL. Zhang, BL. Jue, X. Zhao, and SH. Yan conducted the experiments. RQ. Guo, XL. Sun, and SY. Wang analyzed the data. RQ. Guo, XL. Sun, SY. Wang, B. Qin, and BH. Yue interpreted the results and wrote the article. Y. Jiang, B. Qin, BH. Yue, and MJ. Xue supervised the study. All authors read and approved the final article.

## Declaration of interests

The authors declare no competing interests.

## STAR★Methods

### Key resources table

**Table:** 

REAGENT or RESOURCE	SOURCE	IDENTIFIER
**Antibodies**		

APC anti-human HLA-A,B,C	Biolegend	W6/32, Cat# 311410, RRID:AB_314879
PE anti-human HLA-DR, DP, DQ	Biolegend	Tü39, Cat# 361716, RRID:AB_2750318
FcR Blocking Reagent, human	Miltenyi Biotec	Cat# 130-059-901, RRID:AB_2892112
BV421 Mouse Anti-Human CD3 antibody	BD	SK7, Cat# 563798
PerCP/Cyanine5.5 anti-human CD25 Antibody	Biolegend	BC96, Cat# 302626, RRID:AB_2125478
APC/Cyanine7 anti-human CD62L Antibody	Biolegend	DREG-56, Cat# 304814, RRID:AB_493582
Biotin anti-human CD8 Antibody	Biolegend	SK1, Cat# 344720, RRID:AB_2075392
Anti-Human CD28 antibody, Functional Grade	Fcmacs Biotech.	Cat# FMS-900002
Anti-Human CD3, Functional Grade	Multi Sciences	OKT3, Cat# AH003-500

**Chemicals, peptides, and recombinant proteins**		

Zombie NIR Fixable Viability Kit	Biolegend	Cat# 423106
Zombie Aqua Fixable Viability Kit	Biolegend	Cat# 423102
Brilliant Violet 570™ Streptavidin	Biolegend	Cat# 405227
Elesclomol	MCE	Cat# HY-12040
CuCl_2_	Aladdin	Cat# C106774-25g
PMA	beyotime	Cat# s1819
LPS	Solarbio	Cat# L8880
IFN-γ	absin	Cat# abs04123
2-DG (2-Deoxy-D-glucose)	Selleck	Cat# S4701

**Critical commercial assays**		

Ficoll-Paque PLUS	Cytiva	Cat# 17144003
CELLBANKER 2	Zenoaq	Cat# 11891
DNBelab C Series High-through Single-Cell RNA Library	MGI	Cat# 940-000047-00
Qubit ssDNA Assay Kit	Thermo Fisher	Cat# Q10212
Red blood cell (RBC) lysis buffer	Solarbio	Cat# R1010
cell counting kit-8	Beyotime Biotechnology	Cat# C0038

**Deposited data**		

Raw data for scRNA-seq of PBMCs from HDs and patients with WD	This paper	https://ngdc.cncb.ac.cn/gsa-human, GSA-Human: HRA006240
Processed matrix files	This paper	Figshare (https://doi.org/10.6084/m9.figshare.27977781)

**Experimental models: Cell lines**		

K562	Servicebio	Cat# STCC11501G
CCRF-cem	Servicebio	Cat# STCC10908P
JEKO1	Meisen CTCC	Cat# CTCC-001-0059
KMS11	Meisen CTCC	Cat# CTCC-007-0223
293T	Servicebio	Cat# STCC10301P
NK92-MI	Meisen CTCC	Cat# CTCC-007-0216
Jurkat	Servicebio	Cat# STCC10904P

**Experimental models: Organisms/strains**		

bone marrow cells from patients with MM	This study	N/A
bone marrow cells from patients with AML	This study	N/A
bone marrow cells from patients with MDS	This study	N/A
PBMCs of HD	This study	N/A
PBMCs of patients with WD	This study	N/A

**Recombinant DNA**		

IDH2 overexpression lentiviral vectors	This study	N/A
IDH2^R140Q^ overexpression lentiviral vectors	This study	N/A

**Software and algorithms**		

GraphPad Prism 9.5	GraphPad Software Inc.	RRID:SCR_002798
Seurat R package	Satija et al.	https://satijalab.org/seurat/
FlowJo version 10	Flowjo, L.L.C.	RRID: SCR_008520

**Oligonucleotides**		

Primers for RT-PCR, please see Table S2	This study	N/A

### Experimental model and study participant details

#### Mammalian cell line

NK-92MI cells are maintained in MEMα medium, which is supplemented with 0.2 mM Inositol, 0.1 mM β-mercaptoethanol, 0.02 mM Folic Acid, 12.5% horse serum (HS), 12.5% fetal bovine serum (FBS), and 1% penicillin/streptomycin (P/S). PBMCs, BMMCs, KMS11, RPMI8226, THP-1, CCRF-CEM, and Jurkat cells are cultured in RPMI-1640 medium containing 10% FBS and 1% P/S. Jeko-1 cells are cultured in RPMI-1640 medium supplemented with 20% FBS and 1% P/S. K562 cells are maintained in IMDM medium with 20% FBS and 1% P/S. All cells are incubated at 37°C in a humidified atmosphere containing 5% CO_2_.

### Method details

#### Data collection

This study is a retrospective observational analysis utilizing quantitative data. The data was obtained from the electronic medical record system, covering the period from November 2018 to November 2022. Baseline characteristics, such as age, sex, underlying diseases, and medication history were recorded. Additionally, laboratory test results, including blood routine and biochemical tests, were extracted during hospitalization. Blood routine analysis specifically focused on individuals with hemochromatosis disorders and patients with Wilson's disease, both before and after treatment with penicillamine or sodium dithiopropane sulfonate.

#### Isolation of PBMCs

PB samples anticoagulated with EDTA were obtained from four patients with WD and three HDs at The First Affiliated Hospital of Zhengzhou University (ZZU) (Table S1). This study received approval from the Research and Clinical Trail ethics committee at the First Affiliated Hospital of ZZU. PBMCs were isolated using Ficoll-Paque PLUS (Cat # 17144003, Cytiva). Fresh PBMCs were utilized for scRNA-seq assay, while the remaining PBMCs were frozen using CELLBANKER 2 (Cat # 11891, Zenoaq) and stored at -80°C.

#### Single-cell RNA-sequencing library preparation

The freshly isolated PBMCs were subjected to treatment with ACK buffer and subsequently washed twice with DPBS solution containing 0.04% BSA. The scRNA-seq library was prepared using the DNBelab C Series High-through Single-Cell RNA Library (Cat # 940-000047-00, MGI). The experimental procedures, including droplet encapsulation, emulsion breakage, collection of mRNA captured beads, reverse transcription, cDNA amplification, and purification, were performed strictly following the manufacturer's instruction. The cDNA fragments were fragmented into short fragments ranging from 250 to 400 base pairs. Subsequently, sequencing libraries were prepared with indexing following the manufacturer's protocol. The qualification of these libraries was conducted using the bioanalyzer 2100 (Agilent) in conjunction with the Qubit ssDNA Assay Kit (Cat# Q10212, Thermo Fisher). The DNBSEQ-T7 sequencing platform, developed by MGI Tech, was employed for the pair-end sequencing of all libraries. The gene sequences were acquired through the sequencing reads, which comprised a 30-bp read 1 containing unique molecular identifiers (UMIs) of 10-bp, cell barcode 1 of 10-bp, and cell barcode 2 of 10-bp, along with a 100-bp read 2. The sample index was obtained using 10-bp barcodes.

#### scRNA-seq data processing

We utilized an open-source pipeline (https://github.com/MGI-tech-bioinformatics/DNBelab_C_Series_scRNA-analysis-software) to process the sequencing data. The default parameters were employed to execute sample de-multiplexing, barcode processing, and single-cell 3' unique molecular identifier (UMI) counting. The alignment of processed reads to GRCh38 was performed using STAR (2.5.1b). The DropletUtils tool was utilized to filter out background beads and beads with low UMI counts, employing the "barcodeRanks()" function. PISA was employed to generate individual gene x cell matrices for each library.

#### Quality control, dimensional reduction, and clustering of scRNA-seq data

In downstream analyses, only cells that met the following criteria (as calculated by Seurat) were included. These criteria included >200 and <10000 features, as well as <10% reads mapped to mitochondrial genes. Cells were classified via classical remarkable lineage-specific genes (T cells: *CD3D*, *CD3E*, and *CD3G*; NK cells: *NCAM1*, *FCGR3A*, *KLRF1*, *KLRD1*, and *NKG7*; B cells: *CD19*, *MS4A1*, *IGHD*, *IGHM*, and *IL4R*; plasma cells: *IGHM*, *XBP1*, *JCHAIN*, and *MZB1*; pDC: *IL3RA*, *JCHAIN*, *IRF7*, *TCF4*, *LILRA4*, and *CLEC4C*; CD14^+^ monocytes: *S100A8*, *S100A9*, and *CD14*; CD16^+^ monocytes: *FCGR3A*, *S100A8*, and *S100A9*; DC: *CD1C*, *HLA*-*DPB1*, *HLA*-*DPA1*, *HLA*-*DQA1*, and *ITGAX*).

#### Enrichment scores of biological progresses

For each biological signature, enrichment scores were computed using the “*AddModuleScore*()” function.^77^ Biological signatures included the following: cuproptosis driver, expression of ("*FDX1*", "*LIAS*", "*LIPT1*", "*DLD*", "*DLAT*", "*PDHA1*", "*PDHB*"), cuproptosis inhibitor ("*MTF1*", "*GLS*", "*CDKN2A*"), pentose phosphate pathway (PPP) ("*H6PD*", "*PGLS*", "*PGD*", "*TKT*"), glycolysis ("*SLC2A1*", "*SLC2A3*", "*HK1*", "*HK2*", "*GPI*", "*PFKM*", "*ALDOC*", "*TPI1*", "*GAPDH*", "*PGK1*", "*PGAM1*", "*ENO1*", "*PKM*"), Aerobic glycolysis ("*LDHA*", "*SLC16A3*", "*SLC16A7*"), fatty acid metabolism ("*ACACA*", "*FASN*"), TCA-cycle ("*PC*", "*PDHA1*", "*CS*", "*ACO2*", "*IDH1*", "*IDH2*", "*OGDH*", "*SUCLA2*", "*SUCLG2*", "*SUCLG1*", "*SDHA*", "*SDHB*", "*FH*", "*MDH2*"), glutaminolysis ("*SLC1A5*", "*SLC1A2*", "*SLC1A3*", "*GLUL*", "*GLS*", "*GLS2*", "*ALDH4A1*", "*ALDH18A1*", "*OAT*", "*GLUD1*", "*GLUD2*", "*GOT1*", "*GOT2*", "*BCAT1*"), complex I ("*MT-ND3*", "*MT-ND4*", "*NDUFA2*", "*NDUFA3*", "*NDUFA5*", "*NDUFA6*", "*NDUFA7*", "*NDUFA8*", "*NDUFA9*", "*NDUFA10*", "*NDUFA11*", "*NDUFA12*", "*NDUFA13*", "*NDUFAB1*", "*NDUFC2*", "*NDUFS1*", "*NDUFS2*", "*NDUFS3*", "*NDUFS4*", "*NDUFS5*", "*NDUFS6*", "*NDUFS7*", "*NDUFS8*", "*NDUFV1*", "*NDUFV2*", "*NDUFV3*"), complex II&III ("*SDHA*", "*SDHAF2*", "*COQ9*", "*CYC1*", "*CYCS*", "*MT-CYB*", "*UQCR10*", "*UQCR11*", "*UQCRB*", "*UQCRC1*", "*UQCRC2*", "*UQCRFS1*", "*UQCRH*", "*UQCRQ*"), complex IV ("*COX4I1*", "*COX5A*", "*COX5B*", "*COX6A1*", "*COX6B1*", "*COX7A1*", "*COX7A2*", "*COX7C*", "*COX8A*", "*MT-CO2*", "*MT-CO3*", "*NDUFA4*"), complex V ("*ATP5F1A*", "*ATP5F1B*", "*ATP5F1C*", "*ATP5F1D*", "*ATP5MD*", "*ATP5ME*", "*ATP5MF*", "*ATP5MG*", "*ATP5PB*", "*ATP5PD*", "*ATP5PO*", "*DMAC2L*", "*MT-ATP6*", "*MT-ATP8*", "*ATP5IF1*"), and ETC complex (genesets of complex I, complex II/III, complex IV, and complex V).

#### Functional enrichment analysis of GO-terms and KEGG

The *enrichR* package was utilized to conduct functional enrichment analysis on the significantly differentially expressed genes (DEGs), employing the Gene Ontology (GO) database encompassing molecular function (MF), cellular component (CC), and biological process (BP). Additionally, the *enrichR* package was employed to perform enrichment analysis of KEGG pathways.

#### scRNA-seq analysis of patients with MM, AML, and MDS

The data matrices containing human MM samples were obtained by downloading them from the Gene Expression Omnibus (GEO accession: GSE189460). These matrices consisted of 18 bone marrow samples obtained from patients with AA.^78^ Only cells matching the following criteria (as calculated by Seurat) were included for downstream analyses: >200 and <10000 features, as well as <50% reads mapped to mitochondrial genes. The elimination of batch effects was achieved through the utilization of Package *harmony*. The classification of cells was conducted based on classical lineage-specific genes, as depicted in Figure 5E. The methodology and analysis of targeted scRNA-seq for bone marrow cells obtained from patients with AML or MDS have been extensively elucidated in our prior research.^27^ The raw sequencing data files have been uploaded to the Genomic Sequence Archive (GSA) under the accession number subHRA008486, while the processed data matrix has been uploaded to Figshare (https://doi.org/10.6084/m9.figshare.24039114.v1). Additional information can be obtained from our previous report.^27^

#### Cell viability assay

10,000 cells per 100 μL were seeded in each well in 96-well plates. 100μM elesclomol (MCE) or CuCl_2_ (Aladdin) was dissolved, and a three-fold, 7-point dilution series was prepared in either DMSO or dilute water. 1μL of the diluted CuCl_2_ solutions was added to cell cultures immediately after cells were seeded. Then, 1μL of the diluted elesclomol was added to the media 30 minutes later.

After the cells were incubated for 24 h, 48 h, or 72 h, cell viability was measured using a cell counting kit-8 (Beyotime Biotechnology) according to the manufacturer’s protocol. Briefly, 10 μL of the solvent was added in each well, and the cells were further incubated at 37°C under 5% CO_2_ for 4 h. Absorbance at 450 nm with 630 nm as a reference was measured with a plate reader (SepctraMax Absorbance Reader, Molecular Devices), and the normalized viability was calculated as *Vnor* = (*Aexp* – *Aref*)/( *Acon* – *Aref*), where Vnor is the normalized viability, Aref is the absorbance at 630 nm, and Aexp and Acon are the absorbance at 450 nm from the wells treated with ES and CuCl_2_ or DMSO (negative control), respectively. A representative dose-response curve was plotted, and IC50 was calculated.

#### Flow cytometry

100,000 cells per 1 mL were seeded in each well in 24-well plates. 100 μM elesclomol or CuCl_2_ was dissolved and prepared in either DMSO or dilute water. CuCl_2_ solutions were added to cell cultures immediately after seeding, followed by the addition of elesclomol 30 minutes later. The two final concentrations of elesclomol and CuCl2 were both set at 50 nM and 100 nM. Cells were then incubated for 24 h, 48 h, or 72 h before analyzing the expression of type I HLA and type II HLA using flow cytometry.

Viability dye and antibodies against human HLA-A/B/C and HLA-DR/DP/D were used. After FcR blocking (Miltenyi Biotec), cell suspensions were stained on ice for HLA-A/B/C-APC (Biolegend) and HLA-DR/DP/DQ-PE (Biolegend) for 45 min with prepared buffer (PBS with 2% FBS and 2 mM EDTA). Then, the viability was stained with Zombie NIR™ dye (Biolegend) on ice for 20 min after the cells were washed once with 1 mL PBS. Flow cytometry was performed on a BD cytometer using DIVA software (BD Biosciences) and analyzed using FlowJo version 10.

PBMCs from a healthy donor were separated from the whole blood using Ficoll-Paque regents (Ficoll-Paque PLUS, Cytiva), followed by erythrocytes removal using red blood cell (RBC) lysis buffer (R1010, Solarbio). The cells were then washed twice with PBS, resuspended in RPMI1640, and seeded into each well of a 24-well plate, which was pre-coated with 2 μg/mL anti-CD3 antibody (AH003-500, Multi Sciences) at 37°C for 2h, at a density of 2 × 10^6^ cells/mL, and then added anti-CD28 antibody to cells at 2 μg/mL. Then, the plate was placed in a humidified 37°C, 5% CO_2_ incubator for 2 days. 100,000 THP-1-EGFP cells per 0.5 mL were seeded in each well in 24-well plates. CuCl_2_ solutions were added to cell cultures immediately after seeding, followed by the addition of elesclomol 30 minutes later. The two final concentrations of elesclomol and CuCl2 were both set at 50 nM and 100 nM. THP-1-EGFP cells were then incubated for 24 h, 48 h, before being incubated with activated PBMCs for 24h. Then, the cells were harvested after FcR blocking (Miltenyi Biotec) and stained with CD8-Biotin for 30 min on ice. Then, the cells were stained with CD3-Bv421, CD25-PerCP/Cy5.5, HLA-DR-PE, HLA-ABC-APC, CD62L-APC/Cy7, Streptivin-Brilliant violet 570 for 45 min, washed twice with wash buffer, and stained with Zombie Aqua Fixable Viability dye (Biolegend). A BD cytometer was employed to perform the analysis, with the data subsequently analyzed using FlowJo software.

#### Functions of copper and 2-DG in promoting M1 polarization

1 million cells per 1 mL were seeded in each well in 24-well plates. 100ng/mL PMA were added to cell cultures immediately for 48h, followed by adding 100ng/mL LPS and 20ng/mL IFN-γ for culturing another 48h coupled with 100nM copper, 8mM 2-DG, or different concentrations of copper (1μM, 100μM). Cells were then incubated for 48h before analyzing the expression levels of M1-type macrophage markers (e.g., iNOS, TLR-4, CD80, etc.) by qPCR.

### Quantification and statistical analysis

The data are presented based on at least 3 independent experiments as mean ±SD. Statistical significance was calculated by two-tailed Student’s t test. ∗p < 0.05; ∗∗p < 0.01; ∗∗∗p < 0.001; ∗∗∗∗p < 0.0001; ns, not significant. All of the statistical details can be found in the figure legends.

## Supplemental information

Document S1. Figures S1–S10

Table S1. Information of healthy donors and patients with WD

Table S2. qPCR primers
