# Supplementary material for: A single-cell transcriptomic atlas of immune cells in Wilson disease identifies copper-specific immune regulation
Source: iScience. 2025 Apr 17;28(5):112450. doi: 10.1016/j.isci.2025.112450 (PMC12084004; doi:10.1016/j.isci.2025.112450)
Supplement: Document S1. Figures S1–S10 [file mmc1.pdf]

iScience, Volume *28*

Supplemental information

A single-cell transcriptomic atlas of immune cells in Wilson disease identifies copper-specific immune regulation

Shuya Wang, Xianlei Sun, Qingxuan Xin, Jianxiang Shi, Jin Li, Huilin Zhang, Mengjiao Xue, Fanxiang Yin, Zan Qiu, Xiaoqian Wang, Nannan Sun, Yingmei Li, Yaoyao Chen, Liyan Fu, Chaoqi Li, Shaohua Yan, Xian Zhao, Bolin Jue, Yanxia Gao, Baohong Yue, Bo Qin, Yong Jiang, and Rongqun Guo

Supplemental figures

Supplementary Figure 1

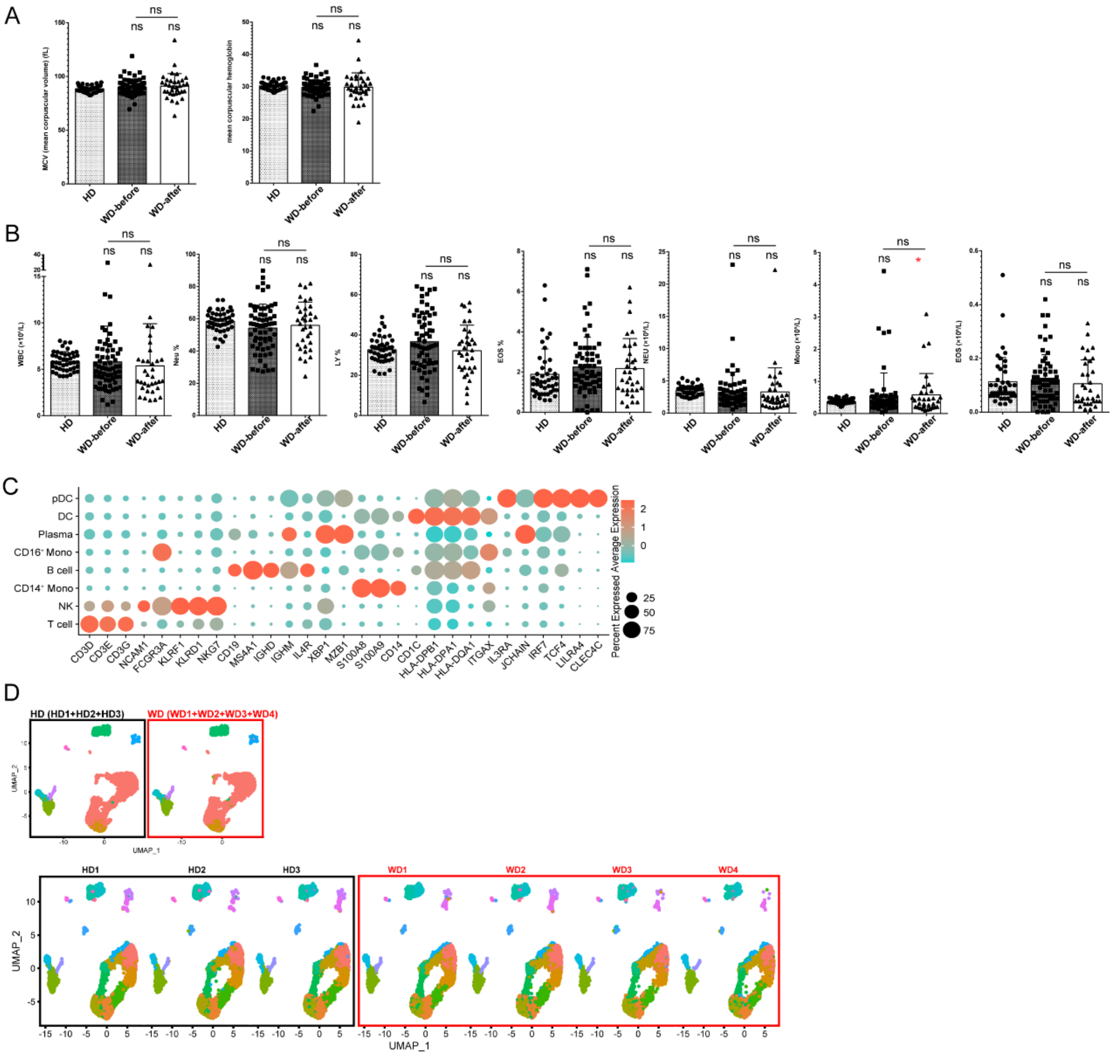

Supplementary Figure 1. Histograms showing the mean corpuscular volume, mean corpuscular hemoglobin **(A)**, white blood cells count, neutrophil count, monocyte count, eosinophil count, proportion of neutrophil, lymphocyte, and eosinophil **(B)** for PB from HD and patients with WD before or after treatment. **(C)** Dot plots showing the lineage-specific marker genes. **(D)** The UMAP plot shows the different distribution of cell subsets in the patients with WD and healthy donors. Patient cells are colorized in the right panel, and healthy control cells are colorized in the left panel.

Supplementary Figure 2

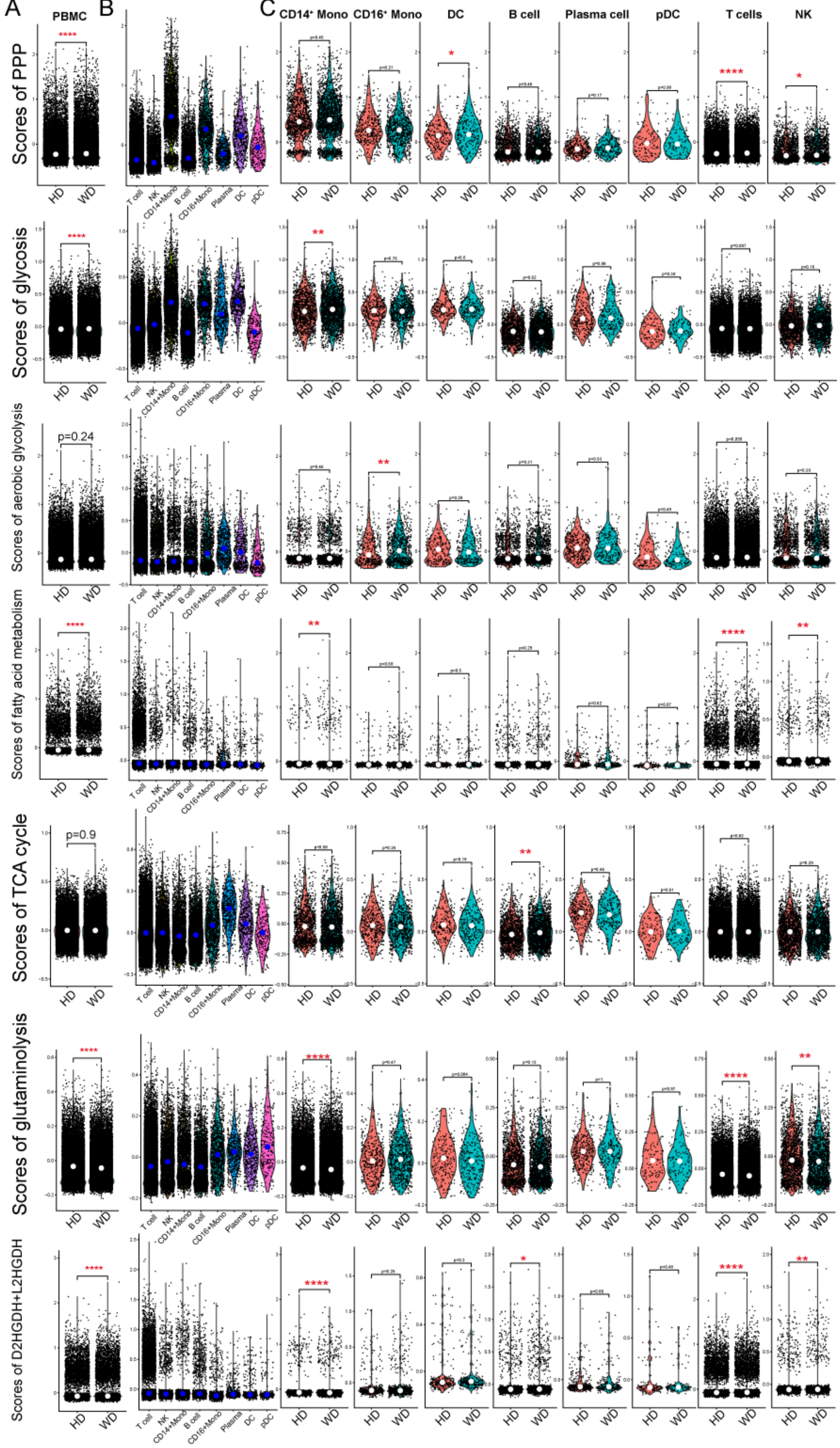

Supplementary Figure 2. The scores of different metabolic pathways in PBMC from different groups **(A)**, different immune subsets **(B)**, and different immune subsets from different groups **(C)**.

Supplementary Figure 3

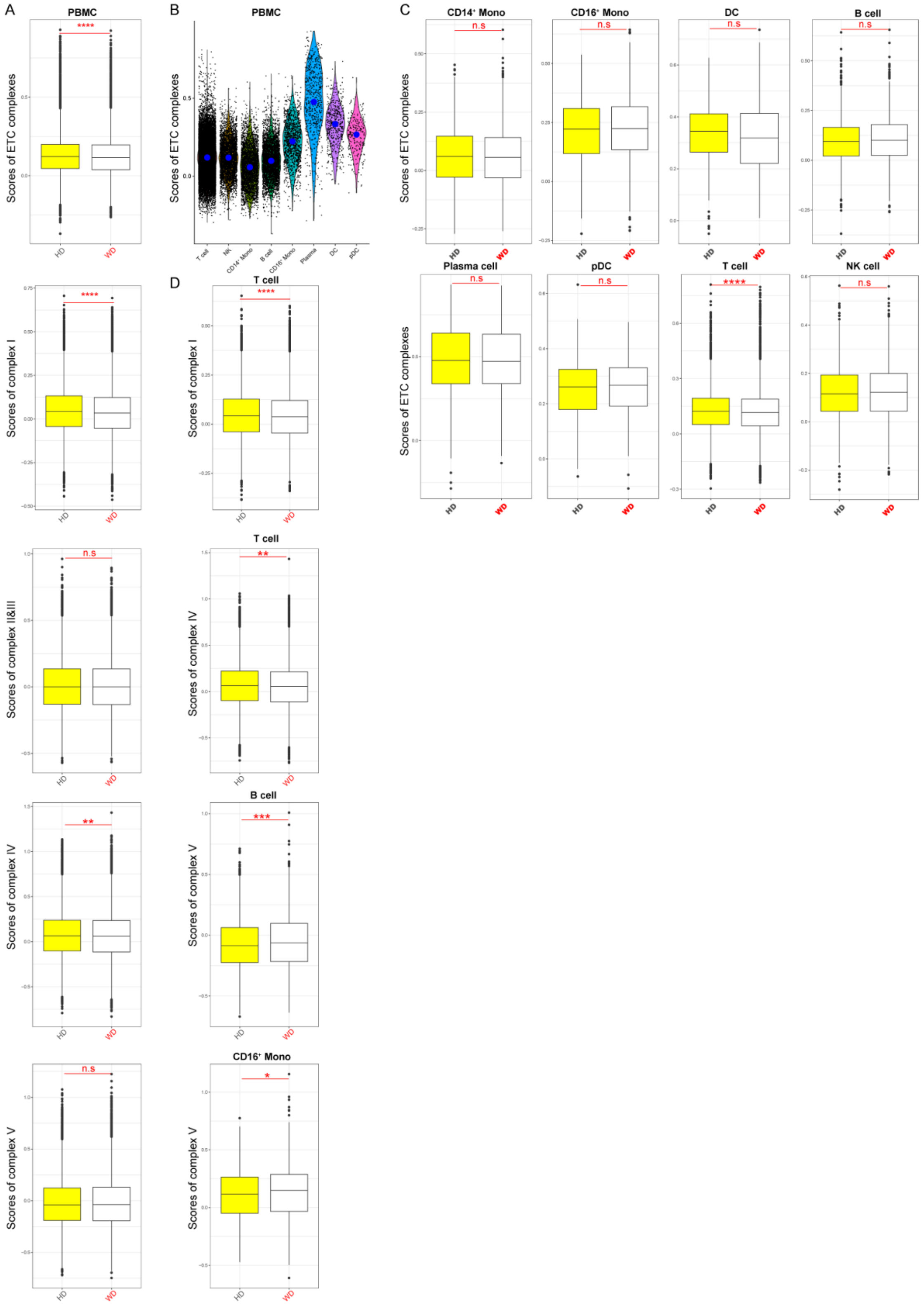

Supplementary Figure 3. The scores of ETC complexes (I, II&III, IV, and IV) in PBMCs from different groups **(A)** and different immune subsets **(B, C)**. The scores of complex V in B cells and CD16^{+} monocytes, and complex I and IV in T cells **(D)**.

Supplementary Figure 4

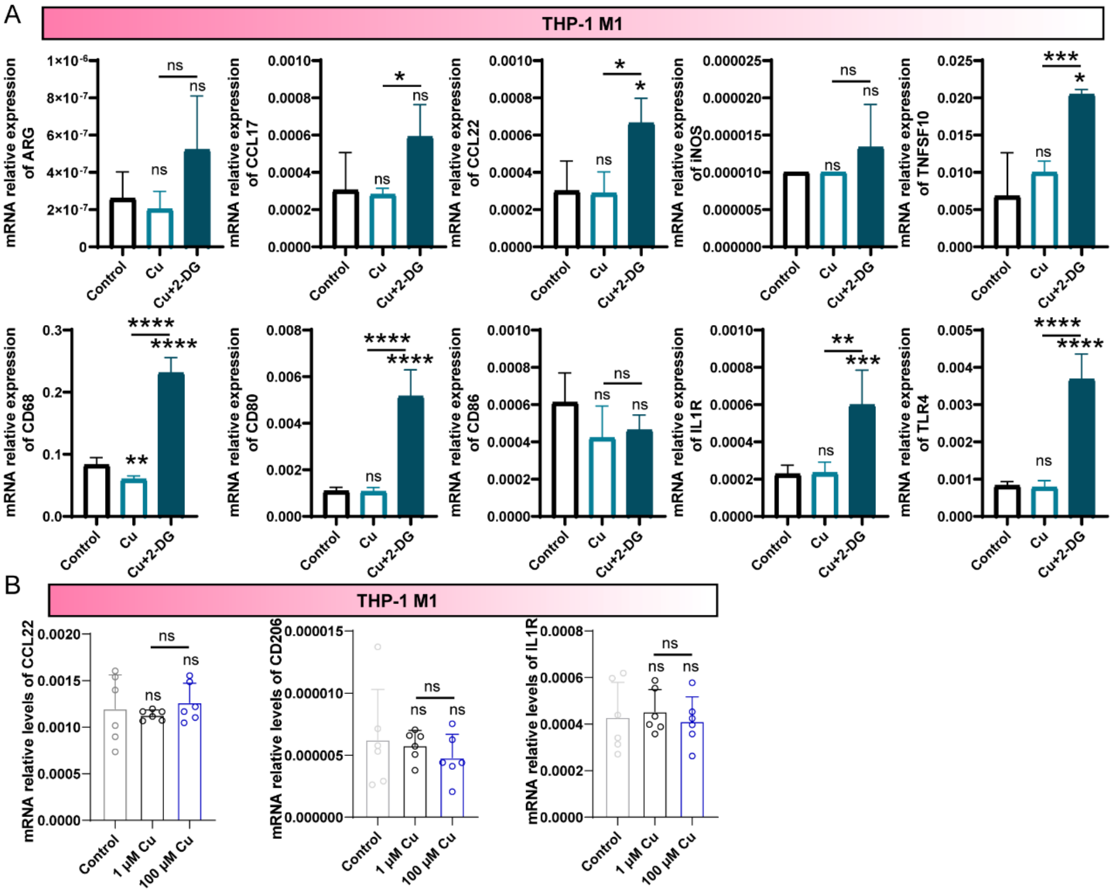

Supplementary Figure 4. IDH2^{R140Q} is a potential therapeutic target of copper-associated agents.**(A)** mRNA levels of *ARG1*, *CCL17*, *CCL22*, *iNOS*, *TNFSF10*, *CD68*, *CD80*, *CD86*, *IL1R*, and *TLR4* in THP-1-derived M1 macrophages after copper (100nM) and 2-DG treatment. **(B)** RT-qPCR was performed to quantify the relative mRNA levels of *CCL22*, *CD206*, and *IL1R* in THP-1-derived M1 macrophages treated with different concentrations of copper.

Supplementary Figure 5

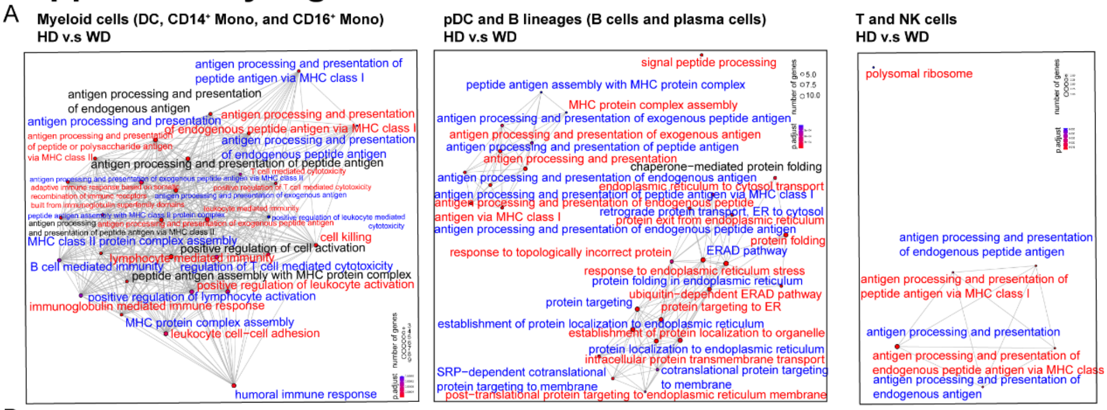

B **Genelist A**

SULT1A1, FOLR3, CFD, RPL3, MYL6, S100A9, CD52, LYZ, HLA-DQB1, HLA-DRB1, SHTN1, LGALS2, S100A8, CES1, ACCS, TAGLN2, PDK4, CCL5, AOAH, MAP3K7CL, NKG7, TRBC2, SIRPB2, CD300E, HLA-DRA, FCER1A, FCER1G, AKAP17A, ADK, SIGLEC10, CRIP1, CAST, TUBA1A, BLVRA, FOS, RNF144B, CRISPLD2, MS4A7, HLA-DPB1, XAF1, TSC22D3, HLA-DQA1

Genelist B

FCRL5, HLA-DRB5, PCNX2, RPS10, RPL6, TMSB4X, MS4A1, AL592183.1, DERL3, GLCCI1, CHI3L2, CD38, ELL2, AQP3, EDEM1, HM13, LMAN1, MAN1A1, JCHAIN, PDIA4, AC009570.2, SPCS3, SEC61A1, CANX, SDF2L1, SPCS1, MT-ND4L, TENT5C, PPIB, ITM2C, FCRLA, XBP1, CALHM6, MZB1, SUB1, SSR3, SLAMF7, TMED9, IFNG-AS1, LINC00926, HSP90B1, TXNDC5, MYDGF, FKBP11, TRAM1, CALR, GAPDH, SEC11C, HSPA5, KDM4B, SEC61B, SSR4, IGLC1, IGHA2, IGLC3, UBE2J1, CCR6, IGLC2, IGHG3, NEAT1, IGHG4, IGHG1, TNFRSF1B, RUNX1, IGHG2, IGKC

Genelist C

MYOM2, CD8A, LAIR2, ZFP36L2, PATL2, GZMH, MAL, RPS26, TRGC1

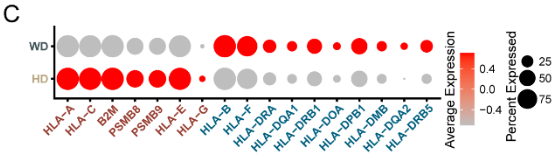

Supplementary Figure 5. The regulation of antigen presenting-associated genes is influenced by an abundance of copper in patients with WD. (A) KEGG analysis of DEGs in myeloid cells, pDC/B lineages, and T/NK cells between HD and patients with WD. **(B)** Lineage-specific DEGs, as supplementary information of Figure 3B. **(C)** Dot plot visualization of HLA-associated genes in PBMCs from different groups (HD and patients with WD).

Supplementary Figure 6

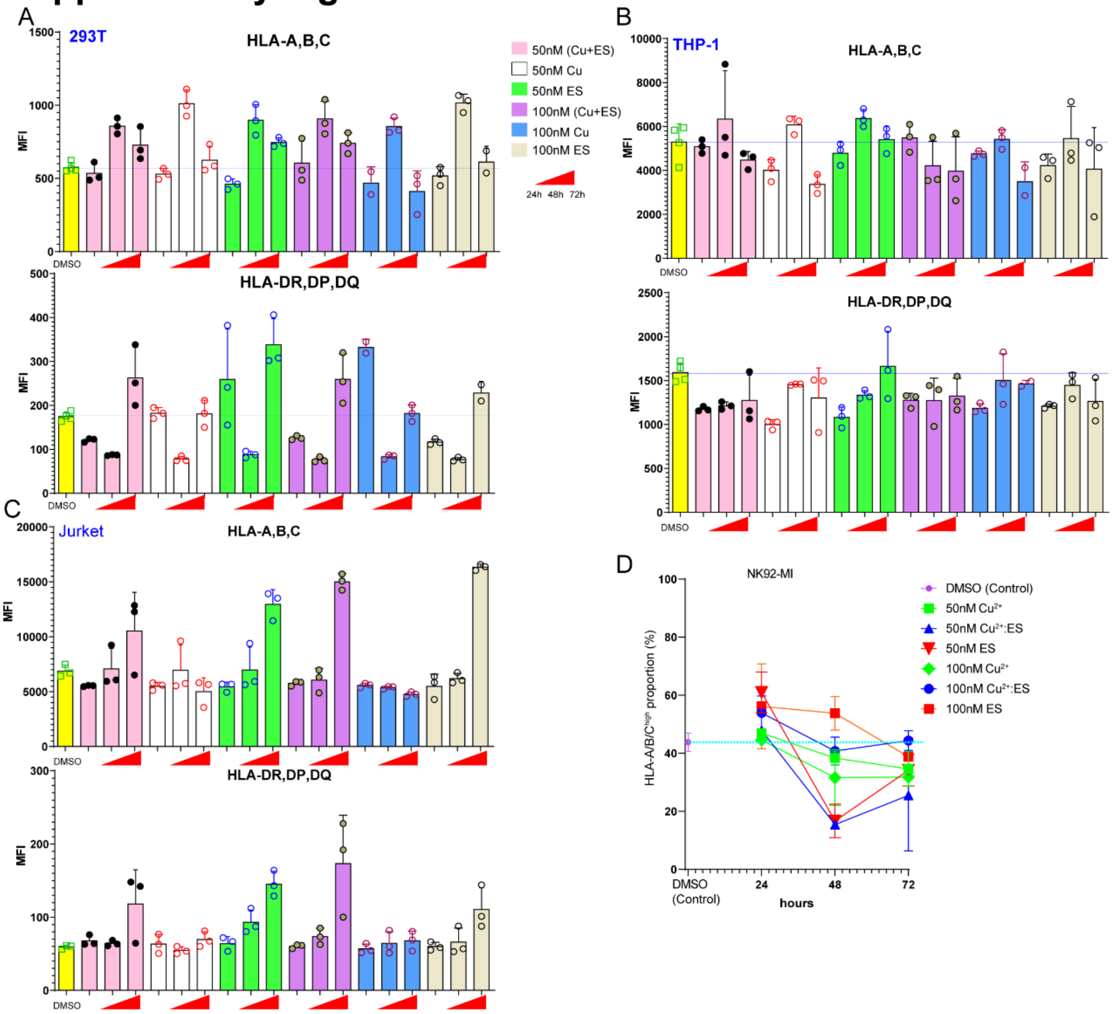

Supplementary Figure 6. The bar diagram shows the MFI for HLA-A,B,C and HLA-DR,DP,DQ expression on 293T cells **(A)**, and Jurket cells **(B)** treatment with the indicated concentrations of copper and/or ES for 24h, 48h, and 72h. Bar graphs show the mean ± SD. **(C)** The proportion of HLA-A, B, C^{high} population on NK92-MI cells treatment with the indicated concentrations of copper and/or ES for 24h, 48h, and 72h. Bar graphs show the mean ± SD.

Supplementary Figure 7

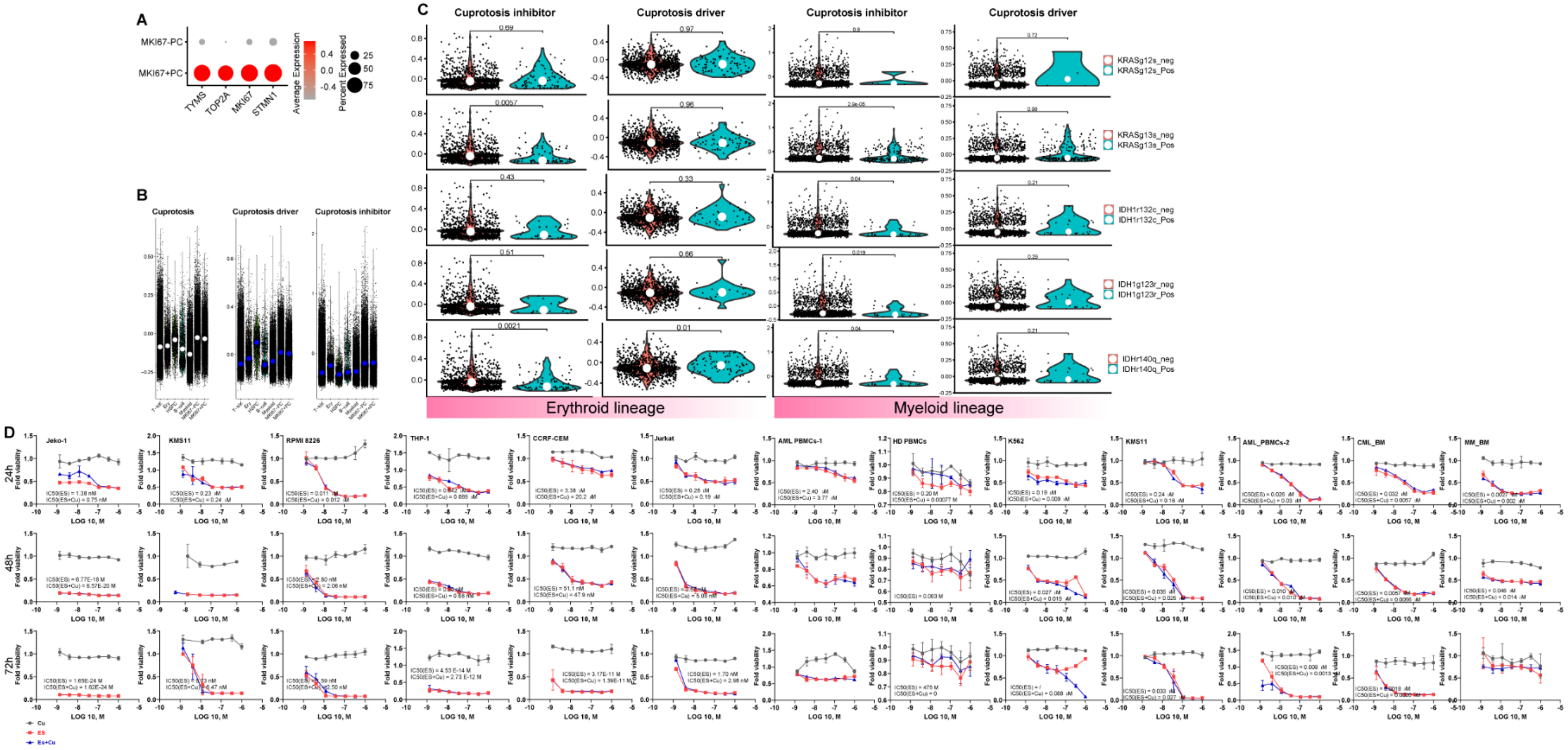

Supplementary Figure 7. (A) Dot plots showing the expression of proliferation-associated genes in plasma cells from HDs and patients with WD. **(B)** The scores of cuprotosis-related genes (cuprotosis inhibitor + driver), cuproptosis inhibitor gene set, and cuproptosis driver gene set in different immune subsets from patients with MM. **(C)** The scores of cuproptosis inhibitor-associated genes, and cuproptosis driver-associated genes in mutation-carrying erythroid lineages and mature myeloid lineages with their counterparts from patients with AML or MDS. **(D)** Quantitation of cell death of primary cells and cell lines after 24, 48, and 72 hours of treatment with ES and copper.

Supplementary Figure 8

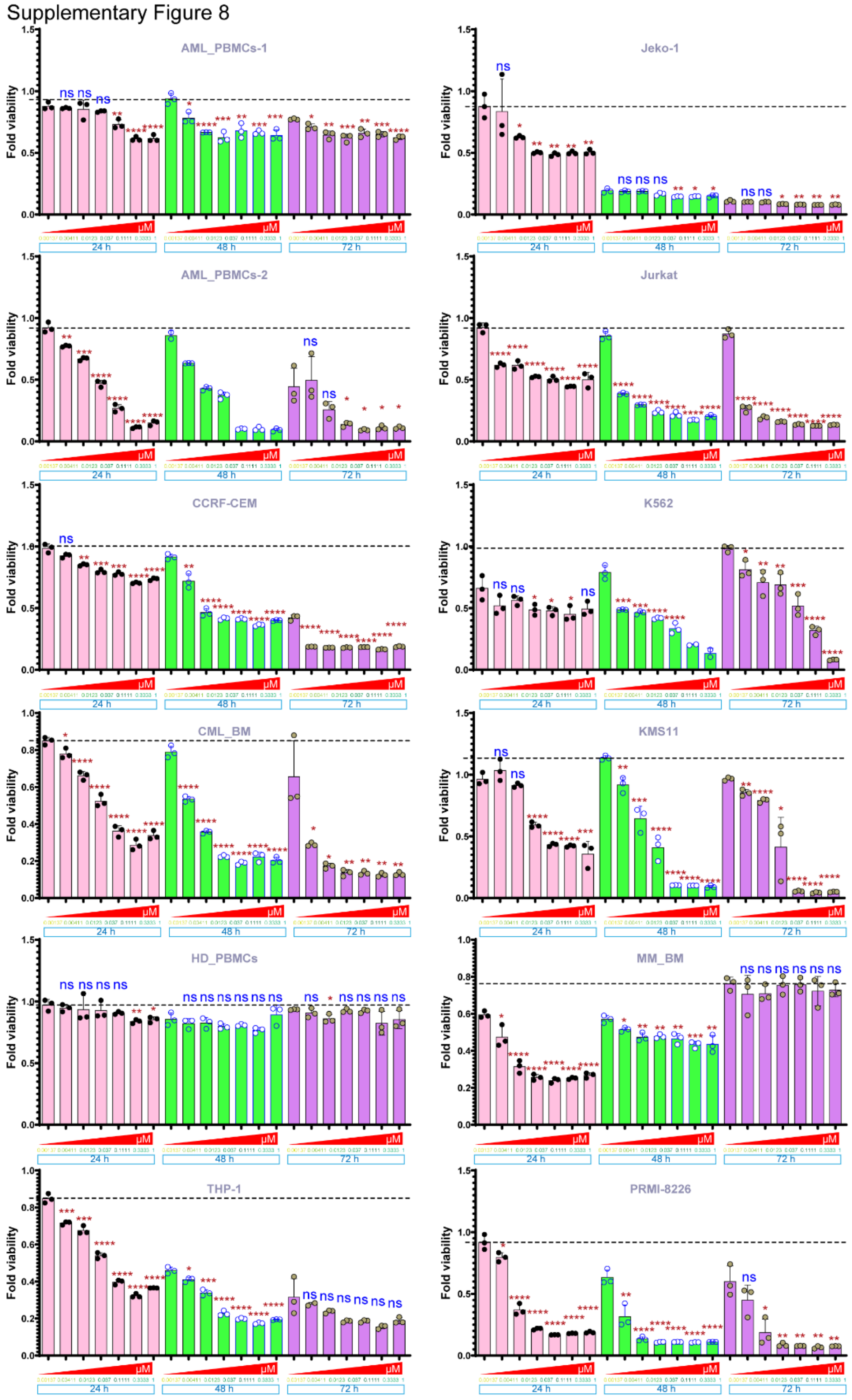

Supplementary Figure 8. (A) Quantitation of cell death of primary cells and cell lines after 24, 48, and 72 hours of treatment with ES and copper.

Supplementary Figure 9

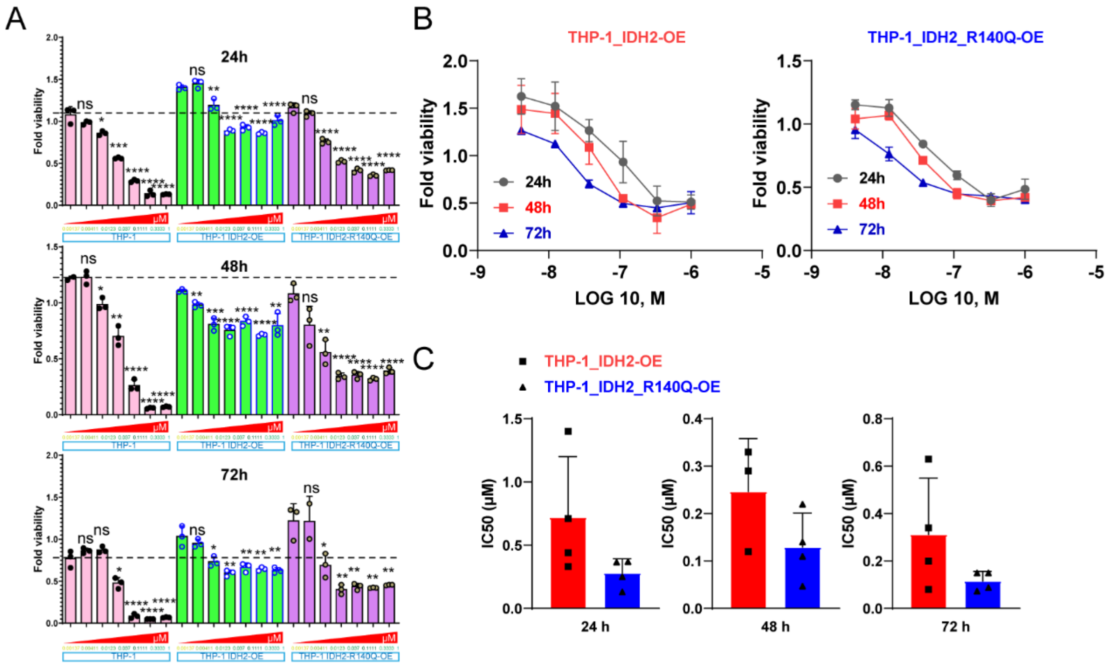

Supplementary Figure 9. (A) Quantitation of alive cells of wild type THP-1, THP-1-IDH2^{OE}, and THP-1-IDH2$^{R140Q-OE}$ cell lines after 24, 48, and 72 hours of treatment with copper. **(B)** An additional experiment was performed for quantitation of cell death of THP-1-IDH2OE and THP-1-IDH2R140Q-OE cell lines after 24, 48, and 72 hours of treatment with copper. **(C)** Bar charts illustrating the IC50 values for two types of cells in different timepoints after treatment.

Supplementary Figure 10

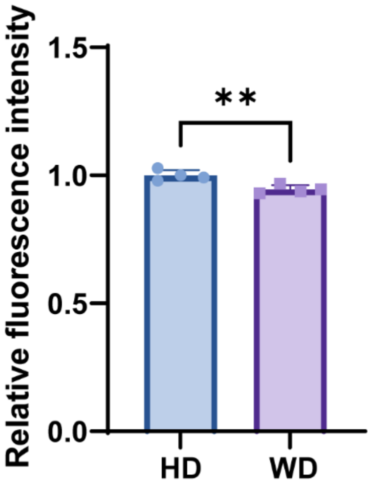

Supplementary Figure 10. The copper in PBMCs from HDs and patients with WD was measured using CuprosGreen (Cat. # TE-98725W, Chinxbt). PBMCs (1×10^5 cells/well) were seeded onto a 96-well plate and treated with GuprosGreen probe solution for 3 hours prior to detecting fluorescence signals with multifunctional microplate reader.
